# Effect of IL-6 receptor blockade on high-sensitivity troponin T and NT-proBNP in rheumatoid arthritis

**DOI:** 10.1016/j.atherosclerosis.2016.10.016

**Published:** 2016-11

**Authors:** Paul Welsh, Katie Tuckwell, Iain B. McInnes, Naveed Sattar

**Affiliations:** a Institute of Cardiovascular and Medical Sciences, University of Glasgow, Glasgow, UK; b Roche Products Ltd, Welwyn Garden City, UK; c Institute of Infection, Immunity and Inflammation, University of Glasgow, UK

**Keywords:** IL-6, Tocilizumab, Troponin, Natriuretic peptide, Rheumatoid arthritis

## Abstract

**Background and aims:**

Observational associations between inflammation and cardiovascular disease are interesting, but randomised experimental data are lacking. We investigated the effect of the IL-6 receptor blocker tocilizumab on N terminal pro B type natriuretic peptide (NT-proBNP) and high sensitivity troponin T (hsTnT) in rheumatoid arthritis (RA) patients.

**Methods:**

A *post-hoc* study was performed in a subset of patients with moderate to severe RA participating in a randomised controlled trial. The effect of tocilizumab on cardiac biomarkers was determined using stored serum (baseline and 24 weeks) in recipients of tocilizumab (8 mg/kg every 4 weeks plus DMARDs; n = 225) or placebo (every 4 weeks plus DMARDs; n = 132).

**Results:**

Median NT-proBNP and hsTnT concentrations at baseline were 100 pg/ml and 5.7 pg/ml, respectively. NT-proBNP decreased in both study arms (median at 24 weeks 77 pg/ml in the placebo arm, 79 pg/ml in the tocilizumab arm; *p*<0.001 for the decrease in both arms), and decreased to a similar extent comparing study arms (tocilizumab effect: −5.5%, *p*=0.55). hsTnT also decreased in both study arms (median at 24 weeks 3.1 pg/ml in the placebo arm, 4.4 pg/ml in the tocilizumab arm; *p*<0.001 for the decrease in both arms). The extent of the reduction in hsTnT was greater in the placebo group (tocilizumab effect: +23.3%, *p*=0.002). Change in NT-proBNP, but not hsTnT, correlated modestly with change in CRP (r = 0.17, *p*=0.013).

**Conclusions:**

These data argue against a rapid preferential benefit of IL-6 blockade on these specific surrogate markers of cardiovascular risk, but may be consistent with a general cardiovascular benefit of improved RA treatment.

**Clinical trials.gov identifier:**

NCT00106574.

## Highlights

• Tocilizumab treatment was associated with a decrease in NT-proBNP, similar in magnitude to the decrease in the placebo arm.
• Troponin T also decreased in both study arms, but decreased slightly more in the placebo arm.
• These data do not support a rapid preferential benefit of tocilizumab treatment on cardiac biomarkers.
• These data may be consistent with the view that improved treatment of rheumatoid arthritis might reduce cardiovascular disease risk.

## 1 Introduction

Both N terminal pro B type natriuretic peptide (NT-proBNP) and high sensitivity troponin T (hsTnT) are emerging strong biomarkers of future cardiovascular disease (CVD) risk in generally healthy people [1]. People suffering from rheumatoid arthritis (RA) have elevated circulating concentrations of natriuretic peptides and troponins, even after adjusting for a wide range of classical CVD risk factors [2], [3], [4]. The mechanisms by which cardiac biomarkers are elevated in RA patients are unclear [2], [3], [4], [5], but one potential pathway may be that systemic inflammation drives vascular dysfunction and atherosclerosis or indirectly causes cardiac stretch.

The inflammatory hypothesis of CVD has evolved from observations that elevated circulating levels of CRP, or indeed almost any other circulating inflammatory marker, are associated with an increased risk of future CVD events in general population studies, even after adjusting for established classical CVD risk factors [6], [7], [8], [9]. Mendelian randomisation studies on IL-6 receptor genes support a causal role for circulating IL-6 in increasing CVD risk [10], [11]. Under the inflammatory hypothesis of CVD, specific interventions to reduce inflammation may reduce vascular pathophysiology and the associated hard CVD events. Observational data suggests that reducing the inflammatory burden in people with RA improves cardiovascular risk [12], [13], but thus far randomised data are lacking; randomised controlled trials of anti-inflammatory therapies in autoimmune inflammatory conditions have not been designed to investigate intervention effects on hard CVD endpoints [14]. In the published observational data, pharmaco-epidemiological studies suggest specifically that RA patients receiving tumour necrosis factor alpha (TNFα) blockers are at lower risk of CVD [15]. Data from an adalimumab treated cohort study reported lowering of NT-proBNP with treatment, but the study lacked a control arm [16]. Therefore further experiments to test the hypothesis that biologic therapies improve cardiac biomarker profiles in a randomised setting are warranted.

The present study assessed the short-term effect of tociluzimab (relative to placebo) on NT-proBNP and hsTnT, perhaps the strongest circulating biomarkers of CVD risk, in the Tocilizumab in cOmbination With traditional DMARD therapy (TOWARD) phase III double-blind randomized controlled trial.

## 2 Patients and methods

### 2.1 Trial design and participants

TOWARD multinational recruitment was conducted between March 2005 and August 2006, as previously described [17]. The study investigated the efficacy and safety of tocilizumab *versus* placebo in combination with standard therapy in patients with moderate-to-severe RA in whom clinical response to classical disease-modifying antirheumatic drugs (DMARDs) was inadequate. Patients aged at least 18 years with moderate-to severe RA of at least 6 months' duration, with a swollen joint count of ≥6, a tender joint count of ≥8, and a C-reactive protein (CRP) level ≥1 mg/dl or an erythrocyte sedimentation rate (ESR) ≥28 mm/h were enrolled. Treatment with conventional synthetic DMARD therapy was stable for 8 weeks prior to study entry [17]. Exclusion criteria included unsuccessful treatment with a TNFα blocker and any cell-depleting therapy. Tuberculosis screening was managed according to local practice.

A total of 1220 patients were randomized (2:1 ratio) to receive either tocilizumab 8 mg/kg intravenously every 4 weeks or placebo intravenously every 4 weeks for 24 weeks. Patients in both groups remained on stable doses of DMARDs. The study protocol was approved by relevant institutional review boards or ethics committees, and written informed consent was obtained from each patient.

### 2.2 Serum sample analysis

Sufficient paired baseline and week 24 samples were available for 357 patients (225 tocilizumab, 132 placebo) who consented to donate serum bio-repository samples. Samples were assayed for hsTnT and NT-proBNP blinded to treatment allocation and time status. NT-proBNP and hsTnT were measured in a single thaw on an automated clinically validated immunoassay analyser (e411, Roche Diagnostics, Burgess Hill, UK) using the manufacturer's calibrators and quality control reagents. High and low control coefficient of variation for each assay was ≤6.6%.

### 2.3 Statistical analysis

Missing biomarker patterns were investigated by comparing baseline demographics and clinical characteristics.

The distributions of each continuous characteristic were examined by randomised group at baseline and 24 weeks and these were summarised as means (standard deviation [SD]) when normally distributed and median (interquartile range [IQR]) when skewed. Categorical variables were reported as frequencies (percentages). Spearman correlations of baseline and change in NT-proBNP and hsTnT with other biomarkers were tested.

The effect of tocilizumab on NT-proBNP and hsTNT was explored by linear regression, with log-transformation of skewed biomarkers. The effect was estimated by comparing the mean 24 week biomarker concentration in the tocilizumab group with the corresponding mean in the placebo group; this approach adjusted for baseline. The results are presented as ratios of geometric means (with corresponding 95% CIs) by exponentiation of the parameter estimates. The linearity and constant variance assumptions were checked by examining plots of residuals against fitted values. To observe a 25% difference in hsTnT between randomised groups at 6 months, with an α of 5% and a power of 80%, we needed 237 participants in the tocilizumab group and 119 in the placebo group. To observe a 10% difference in NT-proBNP between randomised groups at 6 months, with an α of 5% and a power of 80%, we needed 143 participants in the tocilizumab group and 71 in the placebo group. All analyses were performed in STATA version 13.1. *p*-values were not adjusted for multiple comparisons.

## 3 Results

In an assessment of missing data, of the 1220 original treatment assignments, those with available hsTnT data at both time points were slightly older than those with missing data (54.6 *versus* 52.6 years; *p*=0.013), and were more likely to be white ethnicity (78.7% *versus* 69.3%; *p*<0.001). 31.8% placebo group and 28.0% of the tocilizumab group had hsTnT data available at both timepoints; *p*=0.165. Randomised treatment groups were broadly balanced at baseline for key outcome variables (Table 1).

Median NT-proBNP and hsTnT concentrations were 100 pg/ml (IQR 55, 196) and 5.73 pg/ml (IQR 3.41, 8.16) at baseline, respectively. Baseline NT-proBNP and hsTnT correlated with each other and with age; neither were associated with tender or swollen joint count, but NT-proBNP showed modest correlations with DAS28-ESR, IL-6, and CRP (Table 2).

The change in DAS28-ESR over 24 weeks (in both treatment groups combined) showed no correlation with change in hsTnT (r = 0.024, *p*=0.72) or with change in NT-proBNP (r = −0.02, *p*=0.80). Similarly, change in CRP showed no correlation with change in hsTnT (r = −0.076, *p*=0.25) but did show a modest correlation with change in NT-proBNP (r = 0.17, *p*=0.013).

After 24 weeks, changes in DAS28-ESR and CRP in this patients subset with available samples were consistent with data from the main trial (Table 3). Circulating concentrations of both hsTnT and NT-proBNP fell in both treatment groups. The extent of the decrease in NT-proBNP was substantial, and occurred to a similar extent in both treatment and placebo arms. The extent of the decrease in hsTnT was less pronounced in tocilizumab treated patients than in those in the placebo arm, such that the baseline adjusted effect was 23.3% higher in the tocilizumab arm at 24 weeks (Table 3). This effect was not attenuated following multivariable adjustment for allocation of escape therapy during follow-up, week 24 CRP, or week 24 DAS28-ESR.

**Fig. 1:**
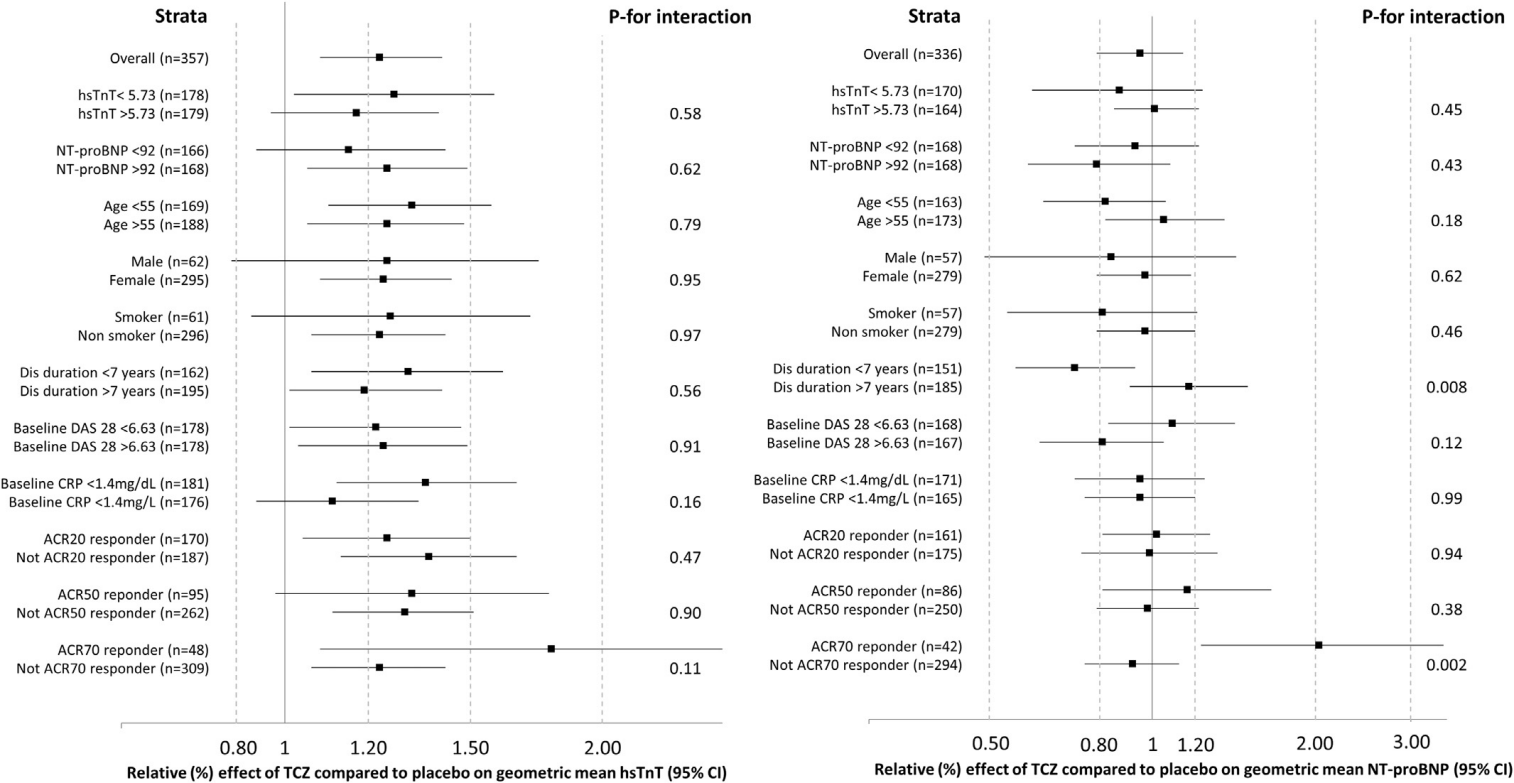
Relative effect of tocilizumab on hsTnT and NT-proBNP stratified by key baseline variables with *p*-values for test for interaction.

Data were further analysed by strata of baseline variables to test for interaction (Fig. 1). There was no strong evidence that the effect of tocilizumab on hsTnT concentration varied by differing levels of baseline variables.

## 4 Discussion

This is one of the first studies to investigate the effect of inflammatory blockade on cardiac biomarkers using a randomised placebo controlled design. In this subset of RA patients there is a clear “trial effect” (also noted in the main trial results [17]) whereby disease activity improved in both arms over 24 weeks, likely due to improved adherence to conventional therapy. It is interesting that the decrease in DAS28-ESR is also accompanied by a decrease in hsTnT and NT-proBNP in both trial arms. However, change in cardiac biomarkers did not correlate with change in DAS28-ESR, although NT proBNP change did correlate modestly with CRP change. Reductions in cardiac biomarkers in both trial arms may be consistent with suggestions that optimally treating disease activity following enrolment into the trial (whatever therapeutic approach is used) will reduce CVD risk [12].

The baseline NT-proBNP levels in these RA patients (median 100 pg/ml) were higher than one might see in a general population; indeed, median NT-proBNP in the ASCOT trial of patients with hypertension and other CVD risk factors was 89 pg/ml [18]. The extent of the reduction in NT-proBNP in both trial arms is fairly substantial. In contrast, the baseline levels of hsTnT were near the limit of detection of the high sensitivity assay, and consequently the absolute reductions in hsTnT are small. One may speculate that the smaller decrease in hsTnT observed in tocilizumab treated patients could be due to a number of mechanisms including release of troponin T from non-cardiac origins; indeed, damaged or regenerating skeletal muscle may express troponin T [19], [20], [21]. RA patients who experience improved quality of life and lower disease activity through tocilizumab allocation might expect to increase muscle mass through increased subsequent physical activity.

In common with data from other studies, the TOWARD trial reported increased total cholesterol levels of around 0.8 mmol/L in those taking tocilizumab [17]. This rise of cholesterol levels while on tocilizumab (which is also common to TNFα blockade targeted biologics [22]) may also provide a potential explanation for the lesser decrease in hsTnT in the tocilizumab arm although the time frame for this change to have biological consequences is short. Of interest, biomarker changes in other pathways appear to be more favourable [23]. Alternatively, type I error may explain our observed differences between arms. Also of importance, we observe decreases in hsTnT in tocilizumab treated patients, despite background rises in cholesterol levels, providing short-term reassurance of tocilizumab being a safe treatment option from a cardiovascular perspective. Evidence that IL-6 blockade is unlikely to cause short-term increases in NT-proBNP, which might be a signal for heart failure risk, in these patients is also reassuring. Endpoint driven trials specifically investigating the inflammatory hypothesis of CVD in secondary prevention patients are underway [24].

One very recent randomised controlled trial of 117 patients investigated the effect of a single dose of tocilizumab on troponin levels during percutaneous coronary intervention; the median AUC for hsTnT during hospitalization over 3 days was 50% higher in the placebo group compared with the tocilizumab group (234 vs. 159 pg/ml, P = 0.007) [25]. The setting of that study is clearly very different from the present one, as it investigated the effect of tocilizumab to reduce inflammation-mediated myocardial injury during a specific intervention. Although the present study provided broadly null results comparing arms, it is based on a larger number of less acutely ill patients over a greater exposure period.

Strengths of the present study include the placebo controlled, randomised, blinded design which offers greater ability to make causal inferences than observational studies. The improvements in cardiac biomarkers that we observe in the placebo arm serve to underline the importance of a control arm. Weaknesses include the availability of samples limited to a subgroup of patients, although this appears to have had little impact on randomised balance. The short term follow-up makes it impossible to speculate as to the long term effect of IL-6 receptor blockade. The reductions in NT-proBNP and hsTnT we observe in both arms of the study over 24 weeks argue against the study being insufficiently powered, having insufficient follow-up, or measurements of hsTnT being too close to the assay limit of detection to observe relevant changes in cardiac biomarkers. Further work, including investigating troponin I levels, as well as upstream markers of CVD risk including endothelial and prothrombotic markers [23], [26], in tocilizumab treated patients is warranted although we could not undertake such work in TOWARD due to limited sample volumes. The main TOWARD trial reported vascular disorders and cardiac disorders as serious adverse events [17]. Vascular disorders occurred in 6.7% of the tocilizumab treated group and 5.1% of the placebo group (*p*=0.25), and cardiac disorders in 0.4% and 0.2% (*p*=0.99) respectively. Given the low event rate and small samples size we could not meaningfully investigate hard endpoints in the present smaller subcohort.

In conclusion, although patients treated with tocilizumab (on top of DMARDS) demonstrated substantial reductions in hsTnT and NT-proBNP, concentrations of hsTnT did not decrease to the same extent as observed in those treated given placebo. These data offer no direct support to the inflammatory hypothesis of CVD, although they also do not rule out a causal role of inflammation in CVD. The data are also perhaps consistent with a general cardiovascular benefit of improved RA treatment, regardless of the drug used. Larger and longer term randomised controlled anti-inflammatory studies are needed to expand on our findings.

## Conflict of interest

PW declares no conflict of interest. KT is an employee of Roche Products and owns share options in Roche. NS has received fees for consulting, speaking, and/or honoraria from Roche, UCB, Merck, Amgen, Sanofi/Regeneron, and Janssen, and IBM from Roche, Pfizer, UCB, Sanofi, Merck, Bristol-Myers Squibb, Janssen, Novartis, Lilly, Abbvie, and Abbott.

## Financial support

Roche provided study samples and a contract of funding for the cardiac biomarker measurements, and subsequent statistical analysis and manuscript preparation, to be conducted at University of Glasgow. PW was supported by a BHF fellowship FS/12/62/29889 during conduct of this work.

## Figures and Tables

**Table 1:** Distribution of baseline variables by randomised treatment group.

Variable	Placebo (n = 132)	Tocilizumab (n = 225)	*p-*value
Age	55.0 (13.5)	54.4 (12.6)	0.640
Female sex	106 (80.3%)	189 (84.0%)	0.373
White ethnicity	101 (76.5%)	180 (80.0%)	0.302
Current smoker	23 (17.4%)	38 (16.89%)	0.897
Disease duration (years)	7 (2, 12)	8 (3, 16)	0.21
DAS28	6.59 (1.06)	6.63 (0.97)	0.683
Number of previous DMARDS	1 (0, 3)	1 (0, 3)	0.728
Baseline diabetes	14 (10.6%)	18 (8.0%)	0.445
C-reactive protein (mg/dl)	1.28 (0.63, 3.19)	1.42 (0.61, 3.03)	0.830
IL-6 (pg/ml)	21.4 (5.4, 46.6)	19.5 (8.3, 46.0)	0.929
Serum creatinine (μmol/L)	66.3 (55.3, 76.9)	62.8 (54.8, 71.6)	0.154
Tender Joint count (n/66)	27.2 (15.3)	30.0 (15.9)	0.10
Swollen joint count (n/68)	18.3 (11.5)	19.6 (12.3)	0.32
Haemoglobin (g/L)	134.7 (15.9)	133.6 (17.0)	0.51
Troponin T (pg/ml)	5.6 (3.1, 8.1)	5.9 (3.6, 8.2)	0.463
NT-proBNP (pg/ml)	92 (55, 209)	105 (54, 185)	0.798

Data are mean (standard deviation), median (inter-quartile range), or n (%).

**Table 2:** Baseline Spearman correlates of NT-proBNP and hsTnT (r-coefficients and associated *p*-values).

	hsTnT	Age	DAS28-ESR	Tender joint count	Swollen joint count	CRP	IL-6	Creatinine	Haemoglobin
NT-proBNP	0.26 (*p*<0.001)	0.36 (*p*<0.001)	0.12 (*p*=0.047)	−0.06 (*p*=0.28)	−0.03 (*p*=0.59)	0.27 (*p*<0.001)	0.22 (*p*<0.001)	−0.01 (*p*=0.89)	−0.24 (*p*<0.001)
hsTnT		0.50 (*p*<0.001)	0.01 (*p*=0.84)	−0.06 (*p*=0.31)	−0.02 (*p*=0.70)	0.00 (*p*=0.96)	−0.03 (*p*=0.58)	0.28 (*p*<0.001)	−0.10 (*p*=0.09)

**Table 3:** Effect of tocilizumab relative to placebo on hsTnT, NT-proBNP, CRP and DAS28 in available samples.

	N pairs	Placebo			Tocilizumab			*p*-value^c^	Baseline adjusted relative effect of tocilizumab
		Baseline Median (IQR)	24 week Median (IQR)	*p*-value^a^	Baseline Median (IQR)	24 week Median (IQR)	*p*-value^b^		
DAS28	104 placebo 208 tocilizumab	6.41 (5.82–7.27)	5.44 (4.58–6.63)	<0.001	6.64 (5.96–7.36)	3.59 (2.37–4.70)	<0.001	<0.001	−42.6% (−35.9%, −48.5%) *p*<0.001
CRP (mg/dl)	106 placebo 213 tocilizumab	1.15 (0.53–2.60)	0.94 (0.55–2.63)	0.31	1.50 (0.62–3.13)	0.04 (0.02–0.09)	<0.001	<0.001	−94.6% (−92.7%, −95.9%) *p*<0.001
hsTnT (pg/ml)	132 placebo 225 tocilizumab	5.6 (3.1–8.1)	3.1 (1.5–5.4)	<0.001	5.9 (3.6–8.2)	4.4 (1.5–7.5)	<0.001	0.013	+23.3% (+8.0%, +40.7%), *p*=0.002
NT-proBNP (pg/ml)	132 placebo 204 tocilizumab	92 (55–209)	77 (46–149)	0.008	105 (54, 185)	79 (42–143)	<0.001	0.59	−5.5% (−21.4%, +13.7%), *p*=0.55

a Comparing baseline and 24 weeks in placebo group.

b Comparing baseline and 24 weeks in tocilizumab group.

c Comparing placebo and tocilizumab groups at 24 weeks.
